# Unveiling Epidemiologic Insights: A Case–Control Study of Congenital Cleft Lip and/or Palate Using Association Rule Mining

**DOI:** 10.1155/bmri/1011204

**Published:** 2026-06-11

**Authors:** Salaheddin Delshad, Seyed Mohsen Laal Mousavi, Maryam Alidadi, Azam Sabahi, Abazar Hajavi, Seyed Mohammad Ayyoubzadeh, Sorayya Rezayi, Leila Shahmoradi

**Affiliations:** ^1^ Charity Foundation for Supporting Children With Congenital Malformation (MOHKAM) Institute, Tehran, Iran; ^2^ Department of Health Information Management and Medical Informatics, School of Allied Medical Sciences, Tehran University of Medical Sciences, Tehran, Iran, tums.ac.ir; ^3^ Department of Health Information Technology and Management, School of Allied Medical Sciences, Shahid Beheshti University of Medical Sciences, Tehran, Iran, sbmu.ac.ir; ^4^ Department of Health Information Technology, Ferdows Faculty of Medical Sciences, Birjand University of Medical Sciences, Birjand, Iran, bums.ac.ir; ^5^ Department of Medical Education, School of Medicine, Tehran University of Medical Sciences, Tehran, Iran, tums.ac.ir; ^6^ Health Information Management Research Center, Tehran University of Medical Sciences, Tehran, Iran, tums.ac.ir; ^7^ Department of Health Information Technology, School of Management and Medical Informatics, Tabriz University of Medical Sciences, Tabriz, Iran, tbzmed.ac.ir

**Keywords:** Apriori algorithm, association rule mining, cleft lip, cleft palate, congenital, epidemiology

## Abstract

**Background:**

Cleft lip and/or palate (CLP) is one of the most prevalent birth abnormalities and is caused by incomplete growth and failure to close components of the face. The objective of this study was to describe the epidemiologic features of patients with congenital CLP and identify risk factors associated with the disorder.

**Methods:**

The study used the Strengthening the Reporting of Observational Studies in Epidemiology (STROBE) checklist for reporting on 1341 CLP patient records from MOHKAM Institute in Iran. We conducted a descriptive analysis on records spanning from September 2006 to May 2023. After applying eligibility criteria, 950 CLP cases and 950 controls with other congenital conditions were included. Data preprocessing removed bias. We then analyzed the preprocessed dataset using descriptive statistics, association rule mining (ARM), and the Apriori algorithm.

**Results:**

The study revealed that 88% of mothers did not use cosmetics during pregnancy. Additionally, 9% of cases had a history of disease during pregnancy, and 6% had a history of drug use. The study also examined the birth order of children and found that the highest number of children with congenital diseases occurred between the first and third children of the family. The age distribution of mothers showed the highest frequency in the age range of 18–24 years in both the case and control groups, whereas the frequency of fathers was highest in the age range of over 41 years. ARM identified five association rules with confidence values exceeding 0.567, indicating moderate associations between combined demographic and geographic variables and CLP occurrence. The rules demonstrated weak‐to‐moderate lift values, suggesting nonrandom associations within the dataset.

**Conclusion:**

Several maternal and familial factors, including pregnancy‐related conditions and parental age, were associated with CLP. Given the multifactorial nature of CLP and its susceptibility to multiple genetic and environmental influences, further large‐scale and multicenter studies are required to validate these associations and explore additional contributing factors.

## 1. Introduction

Congenital anomalies remain a leading cause of neonatal morbidity and mortality worldwide, particularly within the first month of life [1]. Congenital anomalies include both structural and functional birth defects [2, 3]. Birth defects can result in various forms of impairment, including physical and mental conditions [4]. Cleft lip and/or palate (CLP) is a prevalent craniofacial malformation, constituting approximately 65% of head and neck abnormalities [5, 6]. In accordance with ethnicity, gender, and socioeconomic status, the occurrence of CLP exhibits variations in prevalence [7]. A systematic review and meta‐analysis in 2022 determined the occurrence rate of CLP to be 0.3 per 1000 live births [8]. Cleft conditions demonstrate a gender‐based prevalence, occurring more frequently in males than in females [9]. The distribution is as follows: cleft lip occurs in 59.5% of males and 40.5% of females; cleft palate is present in 57.62% of males and 38.58% of females; and instances including both cleft lip and palate comprise 42.38% men and 29.52% females [10].

The etiology of CLP encompasses both genetic and environmental influences, with new research indicating distinct origins for its three subtypes: cleft lip, cleft palate, and combined cleft lip and palate. Scientific and epidemiological studies underscore the substantial influence of environmental risk factors, including as smoking, alcohol intake, prenatal malnutrition, viral infections, teratogenic medications, folate insufficiency, and body mass, on the emergence of various congenital malformations. Environmental factors, in conjunction with genetic predispositions, contribute to the intricate embryonic development resulting in cleft formation [10].

A review study examined the prevalence of CLP in Iran, revealing a rate of 0.34 per 1000 cases, with Tehran recording the highest prevalence and northwest Iran having the lowest occurrence [11]. Factors such as individual biological and environmental risks, along with genetic influences, might be responsible for the elevated prevalence rate [12–14]. Rezq Alswairki et al. conducted recent research and found that maternal passive smoking significantly contributes to the occurrence of CLP [15]. CLP affects patients′ quality of life, and children with CLP face numerous health complications, including hearing loss, speech difficulties, feeding problems, and developmental disorders [16–19]. Patients with CLP frequently experience inadequate saliva production, increased periodontal problems, and oral disorders, often due to maxillary hypoplasia and oral respiration [20–22].

Given the multifactorial etiology and the wide range of associated risk factors, CLP analysis requires the management of complex and high‐dimensional data. Traditional methods for processing and analysis can be inefficient due to the complexity and volume of this data [23]. Data mining (DM), a subfield of knowledge discovery from databases (KDD), is an interdisciplinary, dynamic study subject that extracts high‐level knowledge from low‐level data in the context of large datasets [24]. KDD initially uses DM and machine learning (ML) approaches to retrieve data from databases, identify patterns, and make predictions based on the included data [25, 26]. Healthcare has seen an increase in the utilization of DM techniques to aid in clinical diagnosis and disease prediction [27]. DM has become an essential tool for extracting valuable information from vast amounts of data through the identification of previously unknown patterns [28, 29]. Healthcare DM is the process of sorting enormous amounts of unprocessed medical data and/or well‐organized health records to find and explore any correlations and associations between facts and data parameters to derive important insights, evidence, scientific takeaways, and conclusions that can improve medical practice and/or knowledge [30, 31]. Therefore, without the application of DM methods, it is challenging to obtain useful insights from the exponential growth of data [32, 33].

Due to the multifactorial nature of CLP, data related to this anomaly are highly complex and multidimensional, and their analysis requires the use of advanced analytical methods. Extracting and investigating hidden relationships between these data can help identify more determinants and promote evidence‐based decision‐making. However, to the best of our knowledge, few studies have used association rule mining (ARM) to uncover patterns in CLP data. Therefore, the aim of this study is to identify key patterns and relationships between risk factors and clinical characteristics of patients with CLP using ARM.

## 2. Methods

This retrospective case–control study was reported in accordance with the Strengthening the Reporting of Observational Studies in Epidemiology (STROBE) statement [34].

### 2.1. Dataset

This study utilized data from the charity foundation for supporting children with congenital malformation (MOHKAM) Institute′s registry. We analyzed records of 1341 patients diagnosed with CLP registered between September 2006 and May 2023. The registry employs standardized clinical report forms. Informed consent for the use of medical records in research was obtained from patients′ parents or legal guardians at the time of initial registration. All data were anonymized and obtained with informed consent following the ethical approval (IR.TUMS.MEDICINE.REC.1401.455).

### 2.2. Eligibility Criteria

From the initial dataset, 950 patients with confirmed CLP were included based on the following criteria: (1) diagnosis verified by a pediatrician, (2) availability of complete demographic and clinical records, and (3) Iranian nationality.

The control group consisted of 950 individuals randomly selected from patients with other registered congenital conditions in the same database. These conditions included, but were not limited to, growth hormone deficiency, clubfoot, esophageal atresia, ventricular septal defect, Hirschsprung′s disease, tetralogy of Fallot, hydrocephalus, and hypospadias. All participants were selected from the same registry to ensure comparability of data collection procedures.

### 2.3. Data Bias

This study is based on data obtained from the registry system of the MOHKAM Institute, a Tehran‐based organization with branches across multiple provinces. However, it is imperative to acknowledge the potential limitations arising from the fact that certain patients afflicted with medical conditions may not have sought treatment at the aforementioned charity or may have received care at alternative healthcare facilities, leading to a lack of information pertaining to these patients.

In addition, it is necessary to acknowledge the conceivable presence of errors during the registration process of these data. Missing data were excluded, and statistical techniques were employed to identify and remove outliers. Outliers were defined as data points significantly deviating from the expected range based on interquartile range (IQR) thresholds or visual inspection of distribution plots. These measures were implemented to ameliorate any potential biases and enhance the overall integrity and reliability of the dataset utilized in this study.

So, as a registry‐based study, the findings may be influenced by selection bias due to incomplete population coverage. Additionally, potential inaccuracies in data entry may introduce information bias.

### 2.4. Data Preprocessing

**Figure 1:**
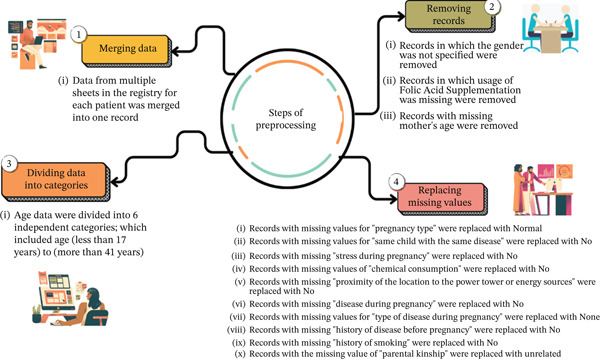
Steps for preprocessing the data.

To clean the extracted medical records, some critical steps were taken into account in the preprocessing stage. In this stage, four main steps are conducted. The following steps were performed to clean the data (Figure 1).

### 2.5. Descriptive Statistics and ARM

**Figure 2:**
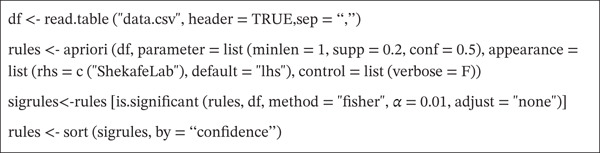
Apriori algorithm in the form of pseudocode.

The preprocessed dataset was imported into RStudio Version 2023.03.0 + 386 (R 4.3.0) for further analysis. Descriptive statistics [35] were analyzed using Microsoft Excel 2019. ARM which is a ML technique used to discover interesting relationships (called association rules) between variables in large datasets [36], specifically the Apriori algorithm, was used to extract association rules related to the presence of congenital CLP. The code used is shown in Figure 2. Our approach involved developing rules in the form of “if‐then” statements, where the “if” part represented patient conditions and the “then” part indicated CLP. To do this, we applied ARM techniques to patient‐related features in patients with CLP. All statistical analyses, including significance testing for rules (via Fisher′s exact test, two‐sided), were performed in R. The threshold for statistical significance was set a priori at *α* = 0.01. We then evaluated the rules using several measures, including support, confidence, and lift, which are commonly used indices in these equations [37, 38]:

(1) Support X⟶CLP=Cased having X and CLPTotal number of cases

(2) Confidence X⟶CLP=Cases having X and CLPCases having X

(3) Lift X⟶CLP=Cases having X and CLP/Cases having XTotal number of cases.

The support metric measures the proportion of total cases in which both the condition (X) and the outcome (e.g., CLP) occur together. It shows how common the rule is in the dataset Equation (1). The confidence metric measures the likelihood that the outcome occurs given that the condition is present. It reflects the accuracy of the rule Equation (2). The lift metric measures the degree of correlation between X and CLP. A lift value of 1 indicates that X has no influence on CLP, whereas a value greater than 1 indicates a positive relationship between X and CLP. Conversely, a value lower than 1 suggests a negative relationship between X and CLP Equation (3).

The coverage metric measures the proportion of cases that satisfy the antecedent part of the rule and is defined as follows Equation (4) [39]:

(4) Coverage X⟶CLP=Cases having XTotal number of cases

The Apriori algorithm is a widely used approach for ARM. The goal of the algorithm is to create frequent item groups that satisfy a user‐specified threshold given a dataset of transactions. The Apriori algorithm considers an item set X of length *k* as frequent if and only if each of its *k* − 1 subsets is also frequent. This approach reduces the search space and allows rule discovery in a computationally feasible time. Figure 2 displays the pseudocode for the applied technique.

In this study, the cutoff threshold was defined based on the five association rules with the highest confidence levels.

## 3. Result

### 3.1. Baseline Characteristics

In our study, we examined 950 cases with congenital CLP (as cases) and 950 cases with other congenital diseases (as controls). In the case group, 555 cases (58%) were male, whereas in the control group, 530 cases (56%) were male.

We found that 88% of the mothers (835 cases) did not use cosmetics during pregnancy. Moreover, 99% of the mothers (939 cases) had no history of chemical use during pregnancy.

Additionally, 9% of the cases (86 mothers) had a history of illness during pregnancy. There was a history of drug use during pregnancy in 6% of cases (57 mothers). Among the parents, 7% (65 cases) lived in close proximity to the power tower.

Sixty percent of the patients (570 cases) gave birth naturally, whereas 40% (380 cases) underwent a cesarean section. For more detailed demographic information on the case and control groups, please refer to Table 1.

**Table 1:** Demographic characteristics.

	Case (*n* = 950)		Control (*n* = 950)	
	Male	Female	Male	Female
Sex (total = 1900)	Frequency (%^a^)	Frequency (%^a^)	Frequency (%^b^)	Frequency (%^b^)
	555 (58%)	395 (42%)	530 (56%)	420 (44%)
	Positive	Negative	Positive	Negative
	Frequency (%)	Frequency (%)	Frequency (%)	Frequency (%)

Cosmetic consumption during pregnancy	115 (12%)	835 (88%)	97 (10%)	853 (90%)
Proximity of the location to the power Tower or energy sources	65 (7%)	885 (93%)	70 (7%)	880 (93%)
History of disease during pregnancy	86 (9%)	864 (91%)	89 (9%)	861 (91%)
Drug use during pregnancy	57 (6%)	893 (94%)	38 (4%)	912 (96%)
Chemical consumption during pregnancy	11 (1%)	939 (99%)	9 (1%)	941 (99%)
Folic acid supplementation	685 (72%)	265 (28%)	691 (73%)	259 (27%)
Family history of cleft lip	27 (3%)	923 (97%)	54 (6%)	896 (94%)
History of smoking	179 (19%)	771 (81%)	197 (21%)	753 (79%)
History of stress during pregnancy	241 (25%)	709 (75%)	264 (28%)	686 (72%)
Type of delivery	Normal	Cesarean	Normal	Cesarean
	Frequency (%)	Frequency (%)	Frequency (%)	Frequency (%)
	570 (60%)	380 (40%)	478 (50%)	472 (50%)
	Normal	IVF	Normal	IVF

Method of pregnancy	Frequency (%)	Frequency (%)	Frequency (%)	Frequency (%)
	947 (99.7%)	3 (0.3%)	940 (99%)	10 (1%)
	Related	Unrelated	Related	Unrelated

Parental kinship	Frequency (%)	Frequency (%)	Frequency (%)	Frequency (%)
	258 (27%)	692 (73%)	339 (36%)	611 (64%)

^a^ ^a^ *Percentage of each group in cases.*

^b^ ^b^ *Percentage of each group in controls.*

**Figure 3:**
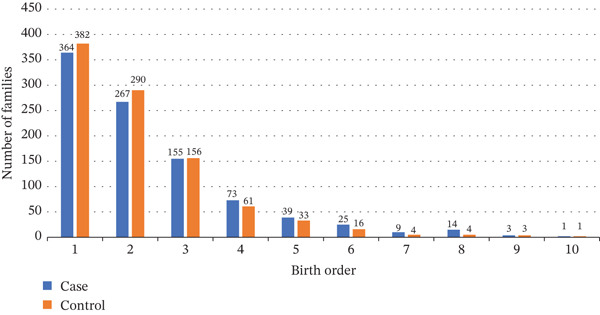
The number of families based on the child′s birth order.

Figure 3 displays the number of families in the case and control groups, sorted by the child′s birth order. The number of children with congenital diseases peaked between the first and third children in the family, whereas the number of children with other congenital diseases (control group) increased until the third child, surpassing the number of children with CLP (case group).

According to the age distribution of mothers and fathers given in Table 2, the frequency of mothers in the age range of 18–24 years was the highest in the case (31%) and control (31%) groups. In contrast, paternal age showed a different pattern, with the highest frequency observed in fathers aged ≥ 41 years in both groups (26% in cases and 29% in controls). This suggests a general shift toward higher paternal age in the study population, although no marked difference was observed between the case and control groups. Overall, no substantial differences in parental age distribution were identified between the two groups, suggesting that age alone may not be a strong discriminating factor for CLP in this dataset.

**Table 2:** Age distribution of mothers and fathers.

Age	Mother		Father	
	Case (%^a^)	Control (%^b^)	Case (%^a^)	Control (%^b^)
≤ 17	29 (3%)	28 (3%)	0 (0%)	0 (0%)
18–24	295 (31%)	291 (31%)	74 (8%)	41 (4%)
25–29	255 (27%)	264 (28%)	156 (16%)	145 (15%)
30–34	190 (20%)	199 (21%)	242 (26%)	233 (25%)
35–40	134 (14%)	125 (13%)	231 (24%)	257 (27%)
≥ 41	47 (5%)	43 (4%)	247 (26%)	274 (29%)
Total	950	950	950	950

^a^ ^a^ *Percentage of each group in cases.*

^b^ ^b^ *Percentage of each group in controls.*

Among patients with CLP, a total of 65 comorbid congenital conditions were identified (Table 3), representing approximately 7% of cases.

**Table 3:** Other congenital diseases associated with congenital CLP patients.

Comorbidity	Frequency (%^a^)	Comorbidity	Frequency (%^a^)
Growth hormone deficiency	8 (1%)	Hirschsprung′s disease	3 (< 1%)
Clubfoot	7 (1%)	Nose deviation	2 (< 1%)
Esophageal atresia	6 (1%)	Tetralogy of Fallot (TOF)	2 (< 1%)
Ventricular septal defect VSD	5 (1%)	Neurogenic bladder	2 (< 1%)
Imperforate anus	5 (1%)	Bladder exstrophy (BE)	1 (< 1%)
Cardiomyopathy	5 (1%)	Omphalocele	1 (< 1%)
Sexual ambiguity	4 (< 1%)	Encephalocele	1 (< 1%)
Scoracratia	4 (< 1%)	Polydactyly	1 (< 1%)
Congenital pelvic dislocation	3 (< 1%)	Biliary atresia	1 (< 1%)
Hydrocephalus	3 (< 1%)	Hypospadias	1 (< 1%)
Total			65 (7%)

^a^ ^a^ *Percentage of each group in cases.*

The most frequently observed associated condition was growth hormone deficiency (1%), followed by clubfoot and esophageal atresia (each 1%). Other congenital anomalies, including ventricular septal defect, imperforate anus, and cardiomyopathy, were also observed at similar low frequencies.

Rare congenital anomalies such as encephalocele, bladder exstrophy, and biliary atresia were observed in isolated cases (< 1%).

Although each comorbidity occurred at a low individual frequency, the presence of a diverse spectrum of congenital anomalies highlights the potential for CLP to co‐occur with multisystem developmental disorders. This pattern may reflect shared embryological pathways or underlying genetic susceptibility affecting multiple organ systems.

Importantly, due to the low frequency of each individual condition, no strong conclusion regarding specific disease clustering can be drawn from univariate description alone, and further multivariable or pattern‐based analyses (e.g., ARM) are required.

**Figure 4:**
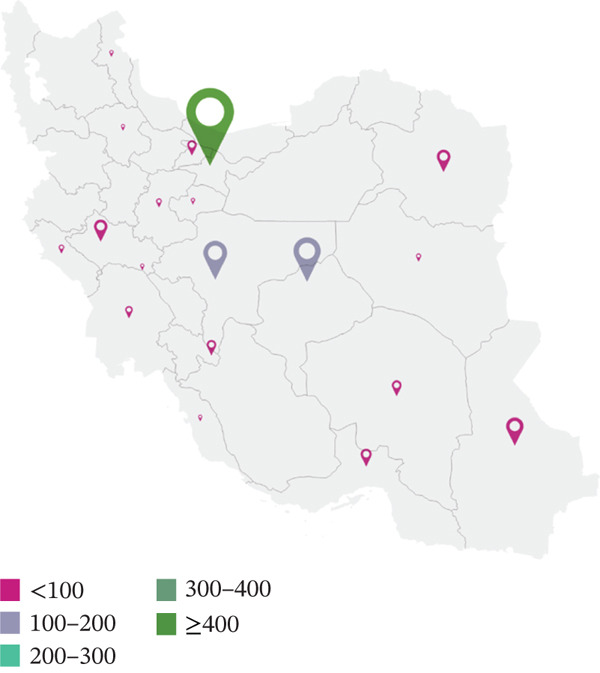
Geographical distribution.

The geographical distribution of study patients is shown in Figure 4. According to geographic distribution data, the highest frequency of congenital CLP disease can be seen in the cities of Tehran, Isfahan, Sistan, and Baluchistan.

### 3.2. ARM Results

Our investigation yielded five rules, with a confidence index surpassing 0.567 (Table 4). The support index values for all the generated rules ranged from 0.203 to 0.285. Additionally, the lift values range from 1.14 to 1.20. Furthermore, the coverage of the rule′s spans from 0.34 to 0.49.

**Table 4:** The rules generated by Apriori method.

	Rules	Consequent	Support	Confidence	Coverage	Lift	Count
1	Geographical distribution: Tehran (True), age distribution of father: 35–40 (False)	{Have disease}	0.202632	0.598756	0.338421	1.198774	385
2	Geographical distribution: Razavi Khorasan (False), type of delivery: normal (True)	{Have disease}	0.284736842	0.582346609	0.488947368	1.165920503	541
3	Geographical distribution: Tehran (True), age distribution of mother: 25–29 (False)	{Have disease}	0.21	0.575757576	0.364736842	1.15272855	399
4	Geographical distribution: Tehran (True), birth order: 3 (False)	{Have disease}	0.224210526	0.574123989	0.390526316	1.149457934	426
5	Geographical distribution: Tehran (True), history of smoking (False)	{Have disease}	0.207894737	0.567528736	0.366315789	1.136253528	395

*In Rule 1, the geographical distribution is Tehran (True), whereas the age distribution of the father is 35–40 (False). {consequential: having a cleft lip}.*

This rule suggests that there is 20.26% support for the occurrence of CLP disease in Tehran, provided that the father′s age is not between 35 and 40. The confidence level was 59.88%, indicating the occurrence of CLP disease. The coverage was 33.84%, which means that this rule applies to 33.84% of the dataset. The lift value of 1.19 indicates a positive correlation between the geographical distribution of Tehran and the father′s distribution: if it falls outside the 35–40 range, it is associated with a higher incidence of CLP disease. A count of 385 signifies the number of cases in the dataset that comply with this rule.

## 4. Discussion

### 4.1. Interpretation of Results

This study applied ARM to identify hidden epidemiological patterns associated with CLP. The findings suggest that CLP is a multifactorial condition influenced by a combination of demographic, environmental, and familial factors rather than isolated exposures.

The present study′s findings demonstrated that more than half of the patients with oral clefts in the case group were male. In this regard, Yilmaz et al. [40] demonstrated that the prevalence of CLP is more common in men than in women (64%). The analysis of other studies also confirmed the findings of the present study [41–43].

Maternal exposure‐related variables such as smoking, drug use, and illness during pregnancy showed modest differences between groups. Although these factors have been widely reported as risk modifiers in previous studies, their relatively small differences in this dataset suggest that their effects may be context dependent and influenced by population‐specific characteristics [44]. Therefore, further research is necessary to pinpoint additional potential factors that could lead to gender differences in oral cleft. It is important to note that additional findings from the current investigation revealed that in both groups, less than 10% of the participants had a history of illness during pregnancy, and almost 5% had a history of drug use during pregnancy. Neves et al. [45] discovered a significant correlation between the mother′s illness, drug use, and chemical exposure throughout the first trimester of pregnancy in their study aimed at identifying environmental elements that may contribute to the development of oral clefts in a Brazilian population. Other studies have reported that drug use during pregnancy increases the risk of oral clefts [46, 47]. However, Spinder et al. [48] concluded in their investigation of the impact of maternal exposure on the occurrence of oral clefts that mothers′ workplace exposure to chemicals such as dust and pesticides enhances the likelihood of oral cleft development in their offspring.

Interestingly, our findings showed that more than 70% of mothers in both the case and control groups reported using folic acid supplements during pregnancy. However, a study by Kelly et al. [49] in Ireland revealed that folic acid supplementation lowers a child′s likelihood of developing oral cleft and neural tube abnormalities. Mendonca et al. [50] conducted a case–control study in India to examine the association between maternal folic acid intake and the risk of nonsyndromic orofacial clefts. There is not a lot of evidence in this study to support the idea that taking folic acid supplements before pregnancy could help prevent nonsyndromic oral clefts [10].

In the present study, 3% of the patients in the case group had a family history of oral clefts. Noorollahian et al. [51] conducted a 10‐year retrospective descriptive study in Iran, using data recorded from 200 patients with oral clefts. The findings of the study indicated that approximately 25% of the patients exhibited a familial predisposition toward CLP, and 63.63% of the patients had parents who were consanguineously married. Furthermore, Jamilian et al.′s study [52] found that a family history of CLP significantly influences the investigation of familial lineage and risk factors among patients with CLP, as well as their accompanying defects. An increased risk of CLP was associated with an odds ratio (OR, 7.4; 95% CI).

In the present study, nearly 20% of the participants in the case group had a history of smoking. In his book *Epidemiology of Cleft Lip and Palate*, Thomson [53] reported that maternal smoking was the only consistent environmental factor associated with CLP. The findings of a comprehensive meta‐analysis comprising 33 scholarly papers by Shi et al. [54] that aimed to determine the association between maternal smoking and environmental tobacco smoke exposure and the risk of orofacial clefts and other birth defects revealed that smoking by mothers was linked to a higher chance of CLP.

Remarkably, in the current study, a quarter of cases in the case group had experienced stress during pregnancy, and more than 25% of cases in both groups were related. In addition, more than a quarter of mothers (31%) in the case group had an age range of 18–24 years, and more than a quarter of fathers (26%) had an age range greater than or equal to 41 years. The systematic review by Inchingolo et al. [55] identified two categories of risk factors for nonsyndromic orofacial clefts: (1) nonmodifiable factors (e.g., genetic polymorphisms, infant sex, race, consanguinity, parental age, socioeconomic status, and geographic/climatic conditions) and (2) modifiable factors (e.g., nutritional status, acute/chronic illnesses, stress, substance use, and environmental exposures). These findings underscore the multifactorial etiology of clefts, with risk profiles varying across populations. This aligns with our study′s emphasis on demographic and environmental determinants in CLP pathogenesis.

Unlike traditional statistical approaches that focus on isolated variables, ARM enabled the identification of multifactorial interaction patterns. The rule linking Tehran residency and paternal age (35–40 years) with CLP suggests that geographic and demographic clustering may jointly contribute to disease occurrence. The findings suggest potential geographic and paternal‐age‐related risk factors, possibly reflecting environmental exposures or genetic predispositions specific to Tehran′s population. However, the observed correlation requires cautious interpretation due to potential confounding by variables such as maternal age, socioeconomic factors, and healthcare accessibility. These results highlight the need for further etiological investigations to elucidate causal mechanisms and assess potential preventive interventions. Some studies have used ARM to discover the signs and patterns of emerging diseases such as COVID‐19 [56] or to identify risk factors for chronic diseases such as cancer [57], cardiovascular diseases [58], or children′s diseases such as early childhood caries [59] and cerebral palsy [60]. The results have shown that this technique can be useful in identifying manifestations and supporting decision‐making in the clinical diagnosis and treatment of diseases. The ARM methodology is a highly effective method for conducting data analysis to identify significant patterns within the context of pediatric critical illness. This technique involves the use of clinical variables and their corresponding numerical values to uncover meaningful associations [61]. Presently, by using DM, physicians can greatly help in investigating the causes and influencing factors in the occurrence of diseases as well as methods of preventing diseases.

### 4.2. Implications for Practice

The findings of our study indicated that comorbidities, residing in densely populated, polluted areas, having a favorable family history, smoking and alcohol use, and having a high maternal age may all be considered risk factors for CLP patients.

As a result, we advise improving fetal period monitoring and supplementing pregnant women′s diets to ensure optimal nutrition.

Identifying the epidemiological characteristics, potential causes, and risk factors of this disease is important for effectively preparing for patients, allocating resources for prevention, diagnosis, treatment, and rehabilitation, and improving the quality of care and life for them.

### 4.3. Strengths, Limitations, and Future Directions

This study represents the application of ARM to analyze a comprehensive set of variables potentially influencing congenital CLP cases. The use of locally sourced data from the MOHKAM Institute in Iran enhances the internal validity and provides valuable epidemiological insights specific to this population. This localized approach strengthens the study′s applicability within the regional context and supports the reliability of the identified associations.

Nonetheless, some limits must be recognized. The data were confined to patients referred to a singular charitable institution, perhaps introducing selection bias and constraining the sample′s representativeness. The findings may lack generalizability beyond the Iranian population due to differences in ethnicity, genetics, socioeconomic level, and environmental factors in other areas. Furthermore, exclusive dependence on the ARM method constrains the analytical scope and may neglect other substantial correlations discernible by different DM or statistical methodologies. These considerations may have diminished the quantity of robust and dependable association rules that could be derived.

Considering these constraints, subsequent research should endeavor to incorporate more diverse and larger populations across many centers and nations to enhance the generalizability of results. Integrating a wider array of DM and ML techniques may reveal supplementary risk variables and connections. Moreover, prospective data collection with stringent quality control could reduce biases associated with absent or erroneous data. These enhanced initiatives will augment comprehension of the epidemiology of congenital CLP and facilitate the development of more effective global preventative and intervention measures.

## 5. Conclusion

Based on the results of the current study, men were more prone to congenital CLP than women were. This finding suggests that gender may be a risk factor for this disease. A proportion of mothers in the CLP group reported a history of illness during pregnancy, and a smaller proportion reported drug use during pregnancy. Although these factors have been previously implicated in congenital anomalies, the observed frequencies in this study should be interpreted with caution in the absence of direct comparison with the control group.

Given that congenital CLP is a complex disorder susceptible to multiple factors, further studies are necessary to validate our findings and explore additional potential risk factors for congenital CLP. Overall, this study provides exploratory insights into the epidemiological patterns of CLP in an Iranian population, which may serve as a basis for hypothesis generation in future research.

## Author Contributions

Seyed Mohsen Laal Mousavi, Abazar Hajavi, Salaheddin Delshad, Seyed Mohammad Ayyoubzadeh, Sorayya Rezayi, and Leila Shahmoradi: conceptualization, methodology, software. Seyed Mohsen Laal Mousavi, Maryam Alidadi, Azam Sabahi, Abazar Hajavi, Salaheddin Delshad, Seyed Mohammad Ayyoubzadeh, Sorayya Rezayi, and Leila Shahmoradi: data curation, writing – original draft preparation. Seyed Mohsen Laal Mousavi, Maryam Alidadi, Azam Sabahi, Abazar Hajavi, Salaheddin Delshad, Seyed Mohammad Ayyoubzadeh, Sorayya Rezayi, and Leila Shahmoradi: visualization, investigation. Abazar Hajavi, Leila Shahmoradi, and Salaheddin Delshad: supervision.

## Funding

No funding was received for this manuscript.

## Disclosure

All authors reviewed and approved the manuscript. The lead author Seyed Mohsen Laal Mousavi affirms that this manuscript is an honest, accurate, and transparent account of the study being reported; that no important aspects of the study have been omitted; and that any discrepancies from the study as planned (and, if relevant, registered) have been explained.

## Ethics Statement

This article results from a study sanctioned by the Students′ Scientific Research Centre of Tehran University of Medical Sciences, designated with the code 1401‐2‐125‐58356. The authors express gratitude to the Charity Foundation for Supporting Children with Congenital Malformation (MOHKAM: https://mohkam.ir/) for their earnest collaboration in supplying the registry system information. The study′s methodology received approval from the Ethics Committee of Tehran University of Medical Sciences (Ethical Approval Number: IR.TUMS.MEDICINE.REC.1401.455). This study is prepared in accordance with the Declaration of Helsinki.

## Consent

The authors have nothing to report.

## Conflicts of Interest

The authors declare no conflicts of interest.

## Supporting information


**Supporting Information** Additional supporting information can be found online in the Supporting Information section. 

## Data Availability

The data that support the findings of this study are not publicly available due to institutional restrictions from TUMS, as the data were obtained under license. However, the data are available from the corresponding author, Seyed Mohsen Laal Mousavi, upon reasonable request and with permission from TUMS.
